# Production and evaluation of *Ighu* from selected cassava varieties using a motorized shredder—a response surface analysis

**DOI:** 10.1002/fsn3.73

**Published:** 2013-11-05

**Authors:** Madu O Iwe, Ann N Agiriga

**Affiliations:** Department of Food Science and Technology, Michael Okpara University of AgricultureUMUDIKE, P.M.B. 7267, Umuahia, Nigeria

**Keywords:** Cassava varieties, *Ighu*, motorized shredder, response surface design

## Abstract

Cassava varieties, TME419, TMS30572, and TMS98/0505, were planted and harvested at 3-month intervals of 10, 13, and 16 months, respectively. A central composite response surface design was used to study the effects of the variables cassava variety, harvesting time, and shredding aperture on selected physicochemical properties of *Ighu* samples. Regression models showed that the experimental variables had significant (*P* ≤ 0.05) effects on the hydrogen cyanide, moisture content, thickness, and width of dry *Ighu*. Minimum values obtainable for the physicochemical properties were 8.1195 mg/kg (10-month, 3-mm shredding aperture from TMS98/0505), 7.58% (13-month, 3-mm shredding aperture from TME419), 0.19 mm (13-month, 3-mm shredding aperture and from TMS30572), and 0.99 mm (16-month, 3-mm shredding aperture from TME419) for hydrogen cyanide, moisture content, thickness, and width, respectively. In addition, *Ighu* produced from 3-mm shredding aperture (TMS30572) at 10-month harvest was the most preferred of all the samples.

## Introduction

Cassava (*Manihot esculenta*), a short-lived perennial plant, stands between 1 and 5 m tall. The main food product is the tuberous roots, which can be retrieved from the soil (Lebot 2009), and it is for these roots that the crop is cultivated (Bradbury and Holloway 1988). The roots deteriorate quickly from the internal heat generated from high respiration rate of the tissue (Ikujenlola and Opawale 2007), high moisture content (Hahn 1989), and subsequent infection and rottening by microbes. This rapid postharvest deterioration means that processing is more important than for any other root crop (Andrew 2002).

Traditionally, cassava roots are processed by various methods into numerous products and utilized in various ways according to the peoples' culture and food habits (Akoroda and Arene 1990; Uzuegbu and Eke 2000). The main products derived from processing cassava are *garri*, *lghu* (*abacha*), cassava flour, *fufu* or *akpu*, and other cassava-based products (Oji 1994). Shredded cassava known variously within the eastern part of Nigeria as *Ighu*, *Nsisa*, *eberebejiapu*/*jigbo*, *mpataka*, *asharasha*, *abacha*, and *jiapummiri* in different *lgbo* dialects (Njoku and Banigo 2006) is a convenient food and a local delicacy. Dry *lghu* is usually soaked in water and eaten as a dessert with coconut, groundnut, palm kernel, dry fish, and meat (Ihekoronye and Ngoddy 1985). It could also be presoaked or partially wetted with water, prepared into a meal with vegetable, palm oil or its emulsion, dry fish, *ugba* (fermented oil bean), onion, and beans as a local salad (Adepoju and Nwangwu 2010). It is usually produced or processed manually using metallic shredding plates, which are moved vigorously by hand on the surface of peeled steamed cassava tubers by a reciprocating action to effect the shredding process or by the use of kitchen knives. Shreds of *Ighu* produced by this local method are not entirely uniform in size, may not be attractive, and the shredding operation is not usually efficient. All these limitations raise the need for mechanization of the shredding process.

Improved processing using a motorized shredder is expected to go a long way toward helping the world to maintain food security. Higher processing efficiency involved in the use of the shredding machine will improve the quality of the product and make them attractive and acceptable in a wider market. Well-dried cassava shreds can be conveniently stored for more than 12 months, making it the most stable cassava product (FAO 1995) and using a motorized shredder will save a lot of labor and time (Etoamaihe 2010). The response surface procedures are a collection involving experimental strategy, mathematical methods, and statistical inference which, when combined, enable the experimenter to make an efficient empirical exploration of the system in which he is interested (Myres and Montgomery 1995). The designs capable of generating a response surface include Central Composite and Box-Behnken designs (Lucas 1994). This study is aimed at producing *Ighu* from selected cassava varieties using a motorized shredder and evaluating the properties of the *Ighu* so produced.

## Materials and Methods

### Source of cassava samples

Cassava roots of TME419, TMS98/0505, and TMS30572 (*Agadagba* species) were obtained from the National Root Crops Research Institute (NRCRI), Umudike, Umuahia Abia State, Nigeria. These roots were monitored from planting and harvested at 10, 13, and 16 months, respectively.

### Preparation of *Ighu*

The cassava varieties were washed and steamed in water for 20 min on a standard 80-cm diameter gas ring and later cooled. When cool, they were peeled. The peeled, steamed cassava tubers were placed into the hopper of the shredding machine and shredded over the shredding plate which has protruding perforations designed to shred the peeled tubers as they slide on top of it because of its reciprocating motion. The shredded tuber strands were discharged beneath the shredding plate to the collection base. Three different shredding disk apertures (3, 6, and 10 mm) of the machine were used for the shredding process. The cassava shreds were soaked in potable water overnight during which time the water was changed twice. They were then thoroughly washed and the washed samples were spread in very thin layers on a flat basket constructed from palm frond material and sundried. The width and thickness of the dry *Ighu* were measured with Vernier calipers and recorded. Three replicates of each test were carried out and the average value was taken. The hydrogen cyanide and moisture content of the *Ighu* samples were determined according to Onwuka (2005).

Sensory evaluation was conducted on the cassava shreds using 20 panelists composed of staff and students of Michael Okpara University of Agriculture, Umudike, Nigeria. The coded samples of *Ighu* produced using the machine were evaluated for appearance, texture (ease of breakage), thickness, and general acceptability using a 9-point Hedonic scale which ranged from 1 (*like extremely*) to 9 (*dislike extremely*), with five as *neither like nor dislike* (Iwe 2010).

### Experimental design and statistical analysis

A face central composite design (*k* = 3) was employed to study the linear, interactive, and quadratic effects of the independent experimental variables. The experimental variables (time of harvesting [10, 13, 16], shredding aperture [3, 6, 10], and cassava varieties [TME419, TMS30572, and TMS98/0505]) were of three levels as stated beside each variable. The center point (0, 0, 0) was replicated nine times. The corner and star points were not replicated (Iwe et al. 1998). The analytical determinations were conducted in duplicate. Data on each run were statistically regressed and analyzed for variance using Minitab software (Minitab lnc., Release 11.21 32bit, State College, PA). Statistical significance was accepted at 5% probability levels (*P* ≤ 0.05). Plots of the fitted significant responses were made using Matlab software (version R2012a) to visualize these effects more clearly (Math Works lnc., Natick, MA).

The Statistical Package for Social Sciences (SPSS—version 20, IBM SPSS Statistics 20, IBM Corporation, Armonk, NY) was used to obtain mean, standard deviation and analysis of variance (ANOVA) was done and judged for significance at *P* ≤ 0.05. Means were separated using Duncan's multiple range test. Optimization was done using the Optimization toolbox of Matlab R2012a software.

## Results and Discussions

The results of the physicochemical properties of dry *Ighu* samples are shown in Table 1.

**Table 1:** Physicochemical properties of dry *Ighu* samples

Run	*X*_1_ (time of harvesting [months])	*X*_2_ (shredding aperture [mm])	*X*_3_ (cassava varieties)	HCN (mg/kg)	Moisture content (%)	Thickness (mm)	Width (mm)
1	10	3	TME419	8.44^d^	7.99^fgh^	0.29^b^	1.09^d^
2	10	3	TMS30572	8.20^e^	8.32^ef^	0.27^b^	1.13^d^
3	10	10	TME419	9.65^ab^	8.59^de^	0.52^a^	5.65^a^
4	10	10	TMS30572	9.73^ab^	9.09^ab^	0.53^a^	5.40^b^
5	16	3	TME419	8.59^d^	7.83^hi^	0.20^b^	1.01^d^
6	16	3	TMS30572	8.88^c^	7.60^i^	0.29^b^	1.05^d^
7	16	10	TME4l9	9.59^b^	8.01^fgh^	0.47^a^	5.32^b^
8	16	10	TMS30572	9.83^a^	8.29^efg^	0.51^a^	5.36^b^
9	10	6	TMS98/0505	8.44^d^	9.21^a^	0.59^a^	4.30^c^
10	16	6	TMS98/0505	8.49^d^	8.99^abc^	0.55^a^	4.25^c^
11	13	3	TMS98/0505	8.20^e^	7.97^gh^	0.22^b^	1.16^d^
12	13	10	TMS98/0505	8.97^c^	8.59^de^	0.52^a^	5.60^a^
13	13	6	TME419	8.50^d^	8.32^ef^	0.51^a^	4.32^c^
14	13	6	TMS30572	8.49^d^	8.78^bcd^	0.52^a^	4.34^c^
15	13	6	TMS98/0505	8.47^d^	8.55^de^	0.54^a^	4.29^c^
16	13	6	TMS98/0505	8.51^d^	8.73^cd^	0.50^a^	4.28^c^
17	13	6	TMS98/0505	8.47^d^	8.59^de^	0.52^a^	4.28^c^
18	13	6	TMS98/0505	8.50^d^	8.71^cd^	0.49^a^	4.20^c^
19	13	6	TMS98/0505	8.53^d^	8.60^de^	0.49^a^	4.20^c^
20	13	6	TMS98/0505	8.49^d^	8.55^de^	0.51^a^	4.28^c^
21	13	6	TMS98/0505	8.48^d^	8.59^de^	0.50^a^	4.27^c^
22	13	6	TMS98/0505	8.46^d^	8.61^de^	0.49^a^	4.31^c^
23	13	6	TMS98/0505	8.48^d^	8.57^de^	0.52^a^	4.20^c^

Means in the same column bearing different superscripts are significantly different (*P* ≤ 0.05).

### Hydrogen cyanide (mg/kg)

There was a significant difference in the HCN content of the dry *Ighu* samples and they varied from 8.20 to 9.83 mg/kg. *Ighu* produced from the cassava varieties TMS30572 10-month, 3-mm shredding aperture and TMS98/0505 13-month, 3-mm shredding aperture had the lowest HCN content while *Ighu* produced from the cassava variety TMS30572 16-month, 10-mm shredding aperture had the highest HCN content. The low value of HCN in the dry *Ighu* samples is brought about by the soaking and the drying processes and the cassava varieties used are improved varieties which have been reported to have low cyanide content (Nweke et al. 1999). Rosling (1987) observed that the cyanide content of food products decreased by drying and processing operations like soaking and cooking. Washing could reduce the HCN content of cassava (Kay et al. 1977). HCN values are lower than the safe level of 10 mg/kg recommended by FAO/WHO (Adindu et al. 2003) and the higher HCN content (9.83 mg/kg) of TMS30572 (10-mm shredding aperture) could be attributed to the short drought experienced at the 16th month of harvest (December). Ernesto et al. (2002) found that flour produced from cassava grown in years with average rainfall in northern Mozambique contained, on average, 40 ppm total cyanide, compared with about 120 ppm in a drought year. Both of these values are well above the World Health Organization safe level of 10 ppm, but the threefold increase in dry years is alarming (Ernesto et al. 2002; Cardoso et al. 2005).

Regression analysis showed that the quadratic effect of shredding aperture and cassava varieties significantly (*P* ≤ 0.05) affected the HCN content of dry *Ighu* samples. And they accounted for 95.3% of the variation in the HCN content of dry *Ighu* samples. ANOVA showed that shredding aperture and interaction between harvesting time and shredding aperture significantly affected the HCN content of the *Ighu* samples.

**Figure 1:**
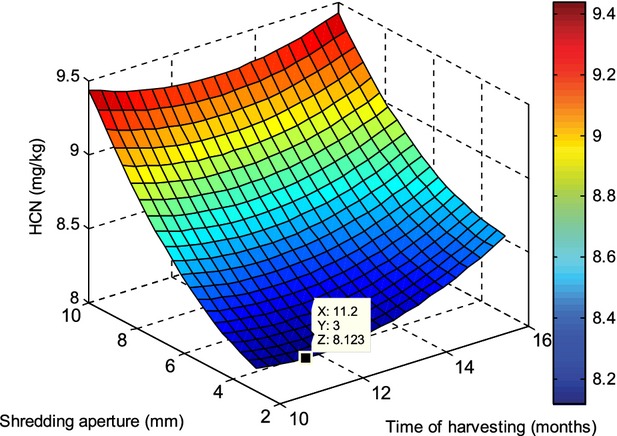
Response surface curves of effects of shredding aperture and time of harvesting on the HCN content of dry *Ighu*.

**Figure 2:**
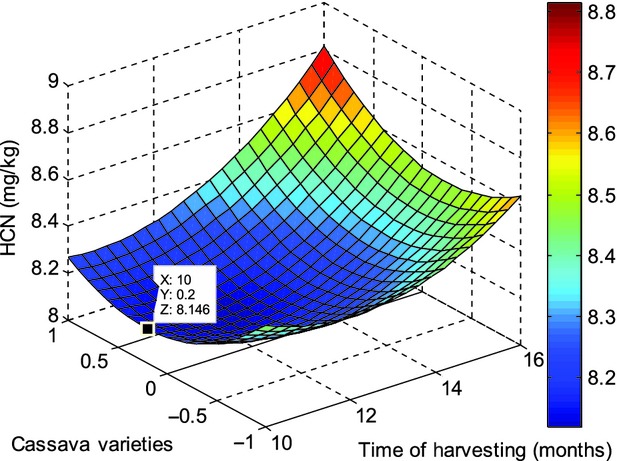
Response surface curves of effects of cassava varieties and time of harvesting on the HCN content of dry *Ighu*.

**Figure 3:**
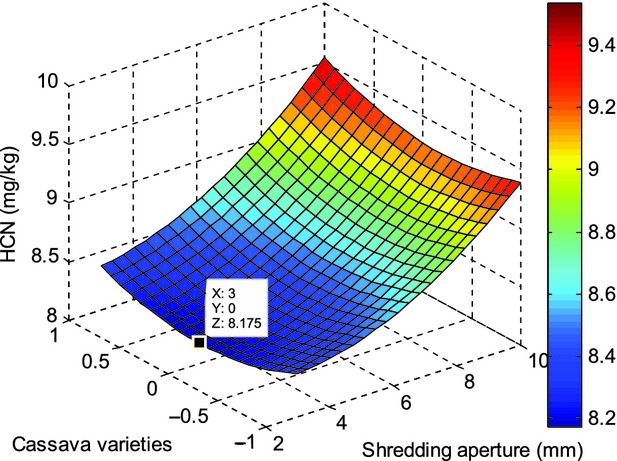
Response surface curves of effects of cassava varieties and shredding aperture on the HCN content of dry *Ighu*.

With reference to optimization, the minimum possible HCN value is 8.1195 mg/kg and to obtain this, the time of harvesting should be 10 months, shredding aperture should be 3 mm, and the choice cassava variety should be TMS98/0505. The response surface curves showing the effects of the experimental variables on the HCN content of dry *Ighu* samples are shown in Figures 1–3.

### Moisture content

Moisture content of the dry *Ighu* samples varied from 7.60% (TMS30572 16-month, 3-mm shredding aperture) to 9.21% (TMS98/0505 10-month, 3-mm shredding aperture). There was a significant difference in the moisture content of the dry *Ighu* samples. Mohammed (2011) reported moisture content of 11.2–12% for dry cassava chips. Processing the cassava tuber into a dry form reduces the moisture content and converts it into a more durable and stable product with less volume which makes it more transportable (IITA 1990; Ugwu 1996). The moisture content of the dry *Ighu* samples was low—less than 10%, making them very stable for storage up to 12 months (FAO 1995).

**Figure 4:**
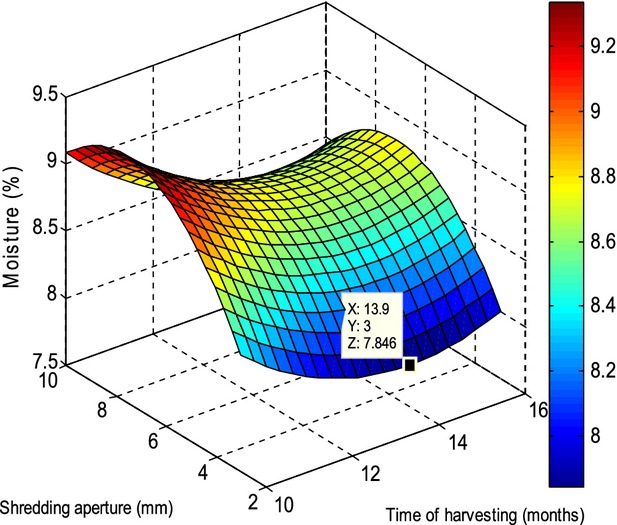
Response surface curves of effects of shredding aperture and time of harvesting on the moisture content of dry *Ighu*.

**Figure 5:**
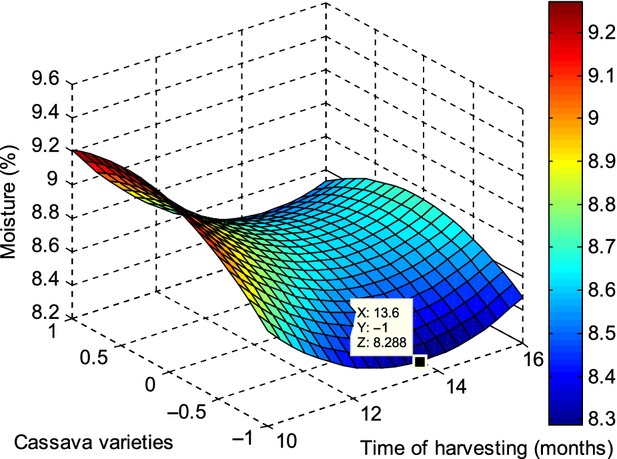
Response surface curves of effects of cassava varieties and time of harvesting on the moisture content of dry *Ighu*.

**Figure 6:**
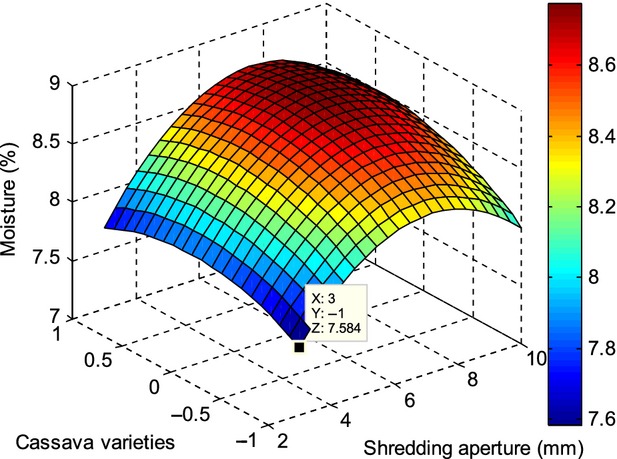
Response surface curves of effects of cassava varieties and shredding aperture on the moisture content of dry *Ighu*.

Regression analysis showed that the linear and quadratic effects of harvesting time and shredding aperture and quadratic effect of cassava variety had a significant effect (*P* ≤ 0.05) on the moisture content of dry *Ighu* samples and these variables accounted for 93.2% variation in the moisture content of dried *Ighu* samples. ANOVA showed that the variables had a significant effect (*P* ≤ 0.05) on the moisture content of the dry *Ighu* samples. Response surface plots of the effects of the variables are shown in Figures 4–6. The plots show that moisture content reduced with a reduction in shredding aperture. The lowest moisture content of 7.58% was obtained when the shredding aperture was 3 mm from cassava variety TME419. However, from optimization, the lowest moisture content obtainable was 7.58%, from the cassava variety TME419 (13-month) shredded with 3-mm shredding aperture.

### Thickness

Cassava variety TME419 16-month, 3-mm shredding aperture had the lowest thickness of 0.20 mm while cassava variety TMS98/0505 10-month, 6-mm shredding aperture had the highest thickness of 0.59 mm. Thickness of the dry *Ighu* samples differed significantly.

**Figure 7:**
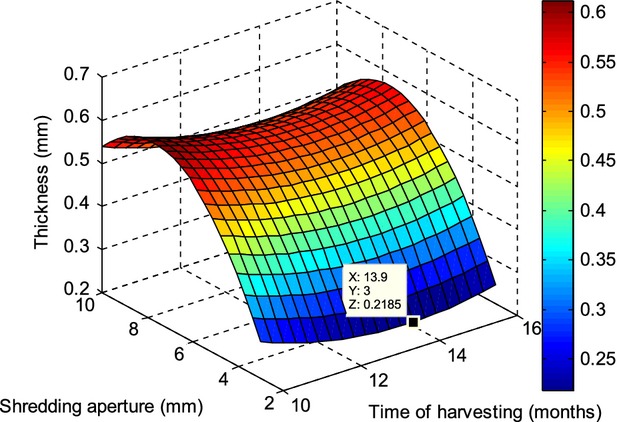
Response surface curves of effects of shredding aperture and time of harvesting on the thickness of dry *Ighu*.

**Figure 8:**
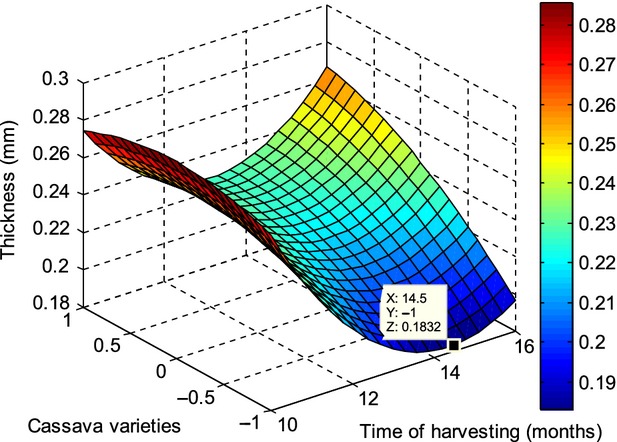
Response surface curves of effects of cassava varieties and time of harvesting on the thickness of dry *Ighu*.

**Figure 9:**
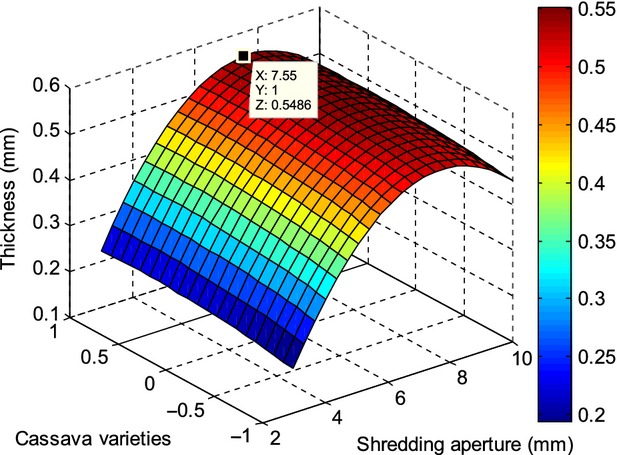
Response surface curves of effects of cassava varieties and shredding aperture on the thickness of dry *Ighu*.

Results of the regression of data on thickness of dry *Ighu* samples showed that the linear effects of harvesting time and interaction between harvesting time and cassava variety were significant (*P* ≤ 0.05) and these accounted for 97.9% variation in the thickness of dry *Ighu* samples. ANOVA showed that all the variables except shredding aperture were significant. Response surface curves of the effects of the variables are shown in Figures 7–9. From optimization, the minimum thickness of dry *Ighu* obtainable was 0.19 mm and it was obtained from 3-mm shredding aperture, cassava variety TMS 30572 and at 13 months of harvest.

### Width

The width of the dry *Ighu* samples ranged from 1.01 mm (TME419 16-month, 3-mm shredding aperture) to 5.65 mm (TME419 10-month, 10-mm shredding aperture). There was a significant difference in the width of the dry *Ighu* samples. It was observed that shredding aperture increased with width of the *Ighu* samples. This is attributable to the larger shredding surfaces which produced larger cassava shreds. The same observation was made by Etoamaihe (2010).

**Figure 10:**
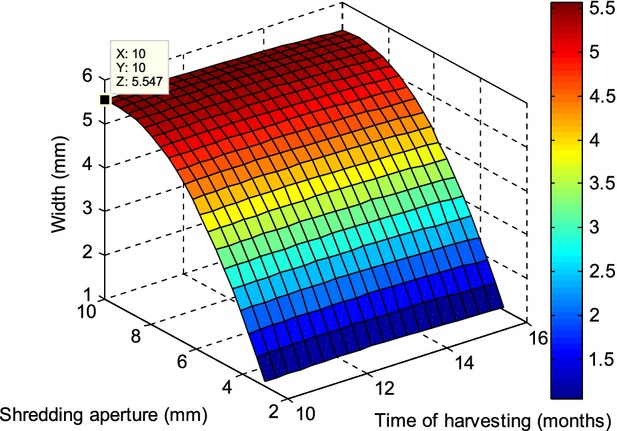
Response surface curves of effects of shredding aperture and time of harvesting on the width of dry *Ighu*.

**Figure 11:**
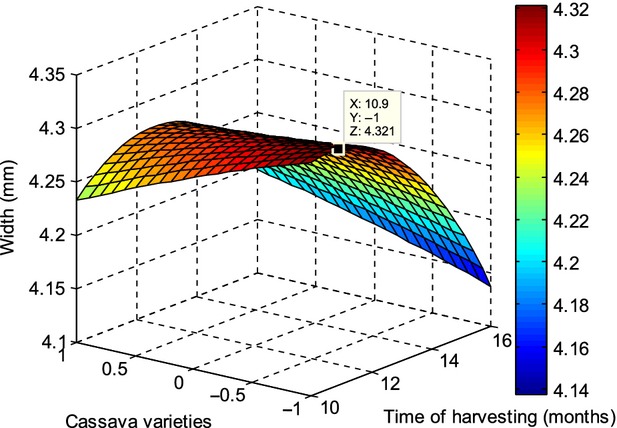
Response surface curves of effects of cassava varieties and time of harvesting on the width of dry *Ighu*.

**Figure 12:**
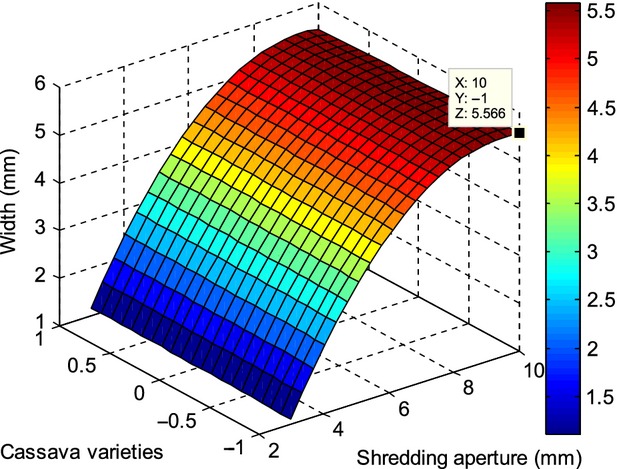
Response surface curves of effects of cassava varieties and shredding aperture on the width of dry *Ighu*.

Regression results showed that the linear and quadratic effects of shredding aperture significantly affected the width of dry *Ighu* samples. From ANOVA, harvesting time and shredding aperture were significant. The response surface curves (Figs. 10–12), confirmed that with the increase in shredding aperture the width of cassava shreds produced by the machine also increased. From optimization, the minimum width of dried cassava shreds obtainable was 0.99 mm and it was obtained at the 16th month from the cassava variety TME419 and 3-mm shredding aperture.

### Sensory evaluation of dry *Ighu* samples

Table 2 shows the sensory evaluation results of dry *Ighu* samples. Cassava variety TMS 30572, harvested at 10 months and shredded with 3-mm aperture, was preferred to all the other samples in all the sensory attributes evaluated. Ekwu et al. (2009) reported that consumers of *abacha*—also known as *ighu*, *mpataka, eberebejiapu*, *nsisa, asharasha*, and *jiapummiri—*do not like thick ones. They observed that it does not enhance effective mixing of the ingredients and condiments used in the preparation of *abacha* meal.

**Table 2:** Effects of processing conditions on the sensory properties of dry *Ighu*

Samples	*X*_1_	*X*_2_	*X*_3_	Appearance	Texture (ease of breakage)	Thickness	General acceptability
1	10	3	TME419	5.45^bcd^	4.25^defg^	4.45^cdef^	4.85^bcde^
2	10	3	TMS30572	2.15^h^	2.60^h^	2.55^g^	1.95^g^
3	10	10	TME419	7.55^a^	6.55^ab^	6.00^ab^	7.25^a^
4	10	10	TMS30572	5.25^bcde^	5.10^bcde^	5.60^bc^	5.35^bcd^
5	16	3	TME419	3.80^efg^	4.15^efg^	3.85^defg^	4.15^cdef^
6	16	3	TMS30572	2.85^gh^	3.50^fgh^	3.70^efg^	3.65^ef^
7	16	10	TME419	5.25^bcde^	4.90^cdef^	5.60^bc^	5.75^b^
8	16	10	TMS30572	5.55^bc^	4.80^cdefg^	5.55^bc^	5.55^bc^
9	10	6	TMS98/0505	5.55^bc^	5.45^abcde^	5.65^bc^	5.90^b^
10	16	6	TMS98/0505	5.15^bcde^	5.20^bcde^	5.45^bc^	5.80^b^
11	13	3	TMS98/0505	3.05^fgh^	3.50^fgh^	3.40^fg^	3.10^fg^
12	13	10	TMS98/0505	7.30^a^	6.70^a^	7.05^a^	7.30^a^
13	13	6	TME419	4.45^bcde^	4.10^efg^	3.60^fg^	4.05^def^
14	13	6	TMS30572	5.75^b^	5.70^abcd^	5.85^abc^	5.50^bc^
15	13	6	TMS98/0505	4.85^bcde^	4.95^cde^	5.10^bcd^	4.75^bcde^
16	13	6	TMS98/0505	4.35^bcdef^	5.25^bcde^	5.05^bcde^	4.80^bcde^
17	13	6	TMS98/0505	4.05^defg^	4.30^defg^	4.55^bcdef^	4.25^cdef^
18	13	6	TMS98/0505	4.15^cdefg^	3.40^gh^	3.45^fg^	4.00^def^
19	13	6	TMS98/0505	5.15^bcde^	5.30^abcde^	5.65^bc^	4.90^bcde^
20	13	6	TMS98/0505	5.45^bcd^	5.90^abc^	5.50^bc^	5.75^b^
21	13	6	TMS98/0505	4.70^bcde^	5.40^abcde^	5.70^bc^	5.75^b^
22	13	6	TMS98/0505	5.80^b^	6.00^abc^	5.25^bc^	5.85^b^
23	13	6	TMS98/0505	5.20^bcde^	5.10^bcde^	4.65^bcdef^	5.55^bc^

Means in the same column bearing different superscripts are significantly different (*P* ≤ 0.05). *X*_1_, time of harvesting (months); *X*_2_, shredding aperture (mm); *X*_3_, cassava variety.

### Appearance

The regression results of data on appearance of dry *Ighu* samples show that cassava varieties and interaction between harvesting time and cassava varieties significantly affected (*P* ≤ 0.05) the appearance of dry *Ighu* samples. Fermentation alters the sensory characteristics of the cassava roots in a way that is often appreciated by local consumers (Ukwuru and Egbonu 2013). Oluwole (2009) reported that physical appearance is an important feature of food products. He added that consumers “eat with their eyes” and use the appearance of foods to predict quality.

**Figure 13:**
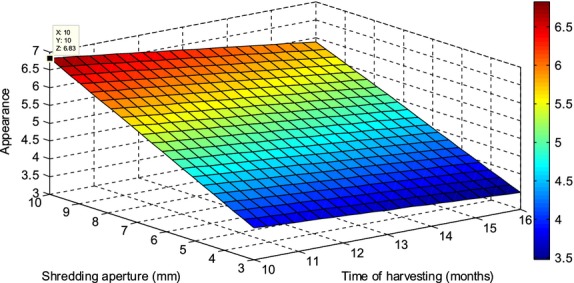
Response surface curve of the effects of harvesting time and shredding aperture on the appearance of dry *Ighu*.

**Figure 14:**
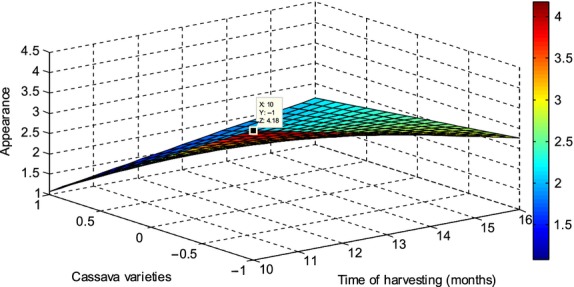
Response surface curve of effects of harvesting time and cassava varieties on the appearance of dry *Ighu*.

**Figure 15:**
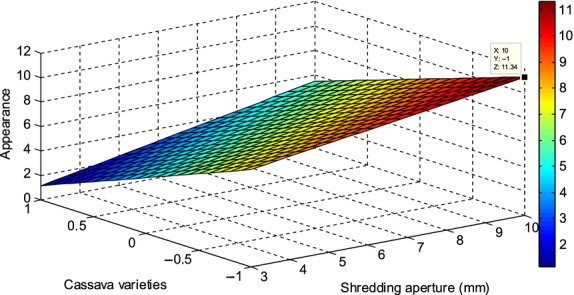
Response surface curve of effects of shredding aperture and cassava varieties on the appearance of dry *Ighu*.

ANOVA showed the effects of the other independent variables to be significant (*P* ≤ 0.05). The studied independent variables accounted for 73.0% of the variation in appearance of the dry *Ighu* samples. Response surface plots of the effects of the variables are shown in Figures 13–15.

### Texture

Regression analysis showed that the experimental variables had no significant effect (*P* ≥ 0.05) on the texture of dry *Ighu* samples. ANOVA showed that the variables had no significant effect (*P* ≥ 0.05) on the texture of dry *Ighu* samples. Texture is measured by the sense of feeling by the skin to determine such textural attributes as crispness, hardness or softness, chewiness, fibrousness, sliminess, etc. (Oluwole 2009). The independent variables accounted for 50% of the variation in the texture of dry *Ighu* samples.

### Thickness

Regression analysis showed that the variables had no significant effect on the thickness of dry *Ighu* samples. ANOVA indicated that the variables had a significant effect on the thickness of dry *Ighu* samples. The variables accounted for 57% of the variation in the thickness of the dry *Ighu* samples.

### General acceptability

From regression analysis, the variables had no significant effect on the general acceptability of dry *Ighu* samples. ANOVA showed that the effects of the independent variables on the general acceptability of dry *Ighu* samples were significant and they accounted for 66% of the variation in the general acceptability of the dry *Ighu* samples. Oluwole (2009) reported that the general/overall acceptability is the combination of all other sensory parameters and that if a product records acceptable quality levels with regard to most of the other parameters, it is expected that such a product will have good overall acceptability.

## Conclusion

*Ighu* was produced from selected cassava varieties harvested at various maturity regimes. There was a significant difference (*P* < 0.05) in the physicochemical and sensory properties of dry *Ighu*. All *Ighu* samples had low HCN content, making them safe for human consumption, and low moisture content, making them suitable for long-term storage. The HCN content of the dry *Ighu* varied from 8.20 to 9.83 mg/kg and regression analysis showed that the quadratic effects of shredding aperture and cassava varieties had significant effects on the HCN content of dry *Ighu*. Optimization showed that minimum HCN obtainable was 8.1195 mg/kg. Moisture content varied from 7.60% to 9.21%. Linear and quadratic effects of harvesting time and shredding aperture and quadratic effect of cassava variety had significant effects on the moisture content of dry *Ighu*. Thickness of dry *Ighu* ranged from 0.20 to 0.59 mm. Regression analysis showed that linear effects of harvesting time and interaction between harvesting time and cassava variety had significant effects on the thickness of dry *Ighu*. Width of dry *Ighu* ranged from 1.01 to 5.65 mm. Width of *Ighu* samples increased with an increase in shredding aperture. Linear and quadratic effects of shredding aperture significantly affected width. Cassava varieties and interaction between harvesting time and cassava varieties significantly affected the appearance of dry *Ighu*. Regression analysis showed that experimental variables had no significant effect on texture, thickness, and general acceptability of dry *Ighu*. In addition to the above data, *Ighu* produced from the cassava variety TMS30572 (10-month) using the 3-mm shredding aperture was the most preferred of all the samples in terms of the sensory attributes evaluated.
